# Structural racism and violence: Routine healthcare access in a cohort of marginalized Indigenous women and Two-Spirit Peoples during the COVID-19 Pandemic

**DOI:** 10.21203/rs.3.rs-3450143/v1

**Published:** 2023-10-31

**Authors:** Chelsey Perry, Shira Goldenberg, Kathleen Deering, Lyana Patrick, Melissa Braschel, Kate Shannon, Brittany Bingham

**Affiliations:** University of British Columbia; San Diego State University; University of British Columbia; Simon Fraser University; University of British Columbia; University of British Columbia; University of British Columbia

## Abstract

**Objectives:**

Historical and ongoing colonial violence, racism, discrimination, criminalization, and intergenerational trauma continues to impact the health of Indigenous women (cisgender and transgender) and Two-Spirit Peoples. Previous and ongoing work clearly articulate the deeply harmful roles of colonialism and racism in continuing to systemically exclude Indigenous Peoples from accessing equitable and culturally safe healthcare. While the COVID-19 pandemic has amplified structural inequities, little attention has been paid to how the pandemic impacts healthcare access for Indigenous women and Two-Spirit Peoples living in urban settings. The aim of this study was to evaluate factors associated with experiencing difficulty accessing routine healthcare in a cohort of marginalized urban Indigenous women and Two-Spirit Peoples on the ancestral, occupied territories of the Musqueam, Squamish and Tsleil-Waututh Nations in what is now referred to as Metro Vancouver, Canada during the COVID-19 pandemic.

**Methods:**

Data were drawn from AMPLIFY, a study of Indigenous cis and trans women and Two-Spirit Peoples in Metro Vancouver. Analyses drew on baseline and semi-annual questionnaire data collected with sex workers and women living with HIV from October 2020–August 2021. We used bivariate and multivariable logistic regression with generalized estimating equations (GEE) to model correlates of experiencing difficulty accessing a family doctor, nurse, or clinic for routine healthcare during the COVID-19 pandemic in the last 6-months.

**Results:**

Amongst 142 marginalized Indigenous women and Two-Spirit Peoples (199 observations), 27.5% reported difficulty accessing routine healthcare. In multivariable GEE logistic regression, participants who had ever been pregnant (AOR:4.71, 95% CI:1.33–16.66) experienced negative changes in psychological and emotional well-being (AOR: 3.99, 95% CI: 1.33–11.98), lacked access to culturally safe health services (AOR:4.67, 95% CI:1.43–15.25), and had concerns regarding safety or violence in their community (AOR:2.72, 95% CI:1.06–6.94) had higher odds of experiencing recent difficulty accessing routine healthcare.

**Discussion:**

Findings are in line with the BC Commissioned In Plain Sight report which recommends the need for accessible, culturally safe, anti-racist, and trauma-informed routine healthcare for marginalized Indigenous cisgender and transgender women and Two-Spirit Peoples during the current and future pandemics. More community-based research is needed to understand access needs for culturally safe routine healthcare amongst marginalized Indigenous cisgender and transgender women and Two-Spirit Peoples.

## Introduction

The COVID-19 pandemic has impacted social and structural health inequities for Indigenous[1] women, Two-Spirit[2] Peoples, and people with marginalized and minoritized sexual and or gender identities, including, lesbian, gay, bisexual, pansexual, asexual, transgender, non-binary, gender fluid, and queer+. Indigenous cisgender and transgender women continue to face multiple layers of ongoing discrimination, criminalization and intergenerational trauma (1). Ongoing colonial violence and gender-based violence continues to impact the health and well-being of Indigenous women, Two-Spirt Peoples, sex workers, and women living with HIV (2–6). Historical and ongoing colonial violence has impacted Indigenous health and Indigenous women Two-Spirit Peoples, women living with HIV, and sex workers face multiple barriers to accessing reliable healthcare services (7) and people with marginalized and minoritized gender identities face increased barriers to accessing healthcare on the ancestral, occupied territories of the Musqueam, Squamish, and Tsleil-Waututh Peoples in what is now referred to as Vancouver (8, 9). Barriers that impact health access are rooted in racism and discrimination. In Canada, racism remains a key determinant of health and significantly impacts Indigenous Peoples access to non-Indigenous-led health services and is well documented in reports such as the British Columbia (BC) Commissioned In Plain Sight report (7, 10, 11). The impacts of the COVID-19 pandemic have left gaps in knowledge that are not only essential to address now but also in preparation for future pandemic planning in the health system. Structural health inequities, including, access to healthcare services and community-based services (10, 12–15), gender-based violence (16, 17), and mental health disparities (14, 18, 19) have been impacted by the COVID-19 pandemic (20), yet little is known about marginalized Indigenous cisgender and transgender women and Two-Spirit Peoples access to routine healthcare services during the pandemic.

The COVID-19 pandemic further magnified existing health inequities that disproportionately impacts the health and well-being of Indigenous women and Two-Spirit Peoples (6, 10, 21). Sex workers and women living with HIV have been affected by the COVID-19 pandemic facing loss of income and lack of access to outreach services as well as stigmatization and harassment by governments and police (22, 23). Gendered impacts of COVID-19 include increased economic insecurity, unplanned pregnancy, lack of access to health services, domestic violence, lack of women’s voice and agency, and mental health issues (6, 12, 15, 24). The impacts are particularly felt by racialized groups that are marginalized by social and structural inequities, including Indigenous women and Two-Spirit Peoples. Due to COVID-19 pandemic lockdowns, mandated social distancing, and fear of the virus, there has been a significant increase in psychological and emotional stress (6, 18, 25–27). Marginalized Indigenous women and Two-Spirit Peoples face intersecting and compounding forms of oppression, and we hypothesize that psychological and emotional stress may further impact healthcare access and utilization. Considering how the intersection of gender and Indigeneity impact access to healthcare, gender diverse[3] and Two-Spirit populations face mental, physical, and sexual health disparities (28) as well as high rates of racism, stigma, and discrimination from healthcare providers that impact their access to healthcare services (24, 29, 30). Sex workers and women living with HIV who identify as a minority gender face increased barriers to accessing healthcare in Vancouver, BC (9, 31). The COVID-19 pandemic has impacted gender diverse populations access to healthcare services and gender-affirming care, including higher rates of violence and victimization (24). Our understanding of access to healthcare and the impacts of the COVID-19 pandemic among Two-Spirit and gender diverse Indigenous Peoples remains limited. Responsive and culturally appropriate research is needed to address the lack of understanding of the health inequities that Two-Spirit and gender diverse Indigenous Peoples face, as well as the reclamation of their healing.

There is conclusive global evidence demonstrating that Indigenous Peoples have worse access to quality healthcare than other populations around the world and Indigenous Peoples face unique barriers to accessing health care services (32–34). It is important to note that research on the health differences between Indigenous and non-Indigenous Peoples does not mean that Indigenous Peoples are inherently more likely to be sick but rather they are experiencing the ongoing impacts of colonial violence, for example, residential schools, the sixties scoop, intergenerational trauma, and ongoing racism (35). Previous and ongoing work clearly articulate the deeply harmful roles of colonialism and racism in continuing to systemically exclude Indigenous Peoples from accessing equitable and culturally safe healthcare. These acts of colonialism have ensured and continue to ensure that Indigenous Peoples are intentionally excluded from accessing equitable healthcare. The impacts of colonial violence on the health of Indigenous Peoples have been severe, leading to health issues that were not present prior to colonization, for example, mental health issues (7), tuberculosis (36), diabetes (37), cancer (38), and violence (22). The widespread racism and violence against Indigenous Peoples in Canadian systems of care led to the *In Plain Sight* inquiry. The *In Plain Sight* report highlights racism as a social determinant of health and Indigenous specific racism as a significant barrier to accessing health services, including accessing a doctor, nurse, and clinic. Racism crucially impacts the health and well-being of Indigenous Peoples in BC (7, 10). Compared to non-Indigenous people, Indigenous Peoples in BC face lower rates of continuity of care and healthcare access (10). Limited action has been taken towards addressing these health inequities that are clearly outlined in the *In Plain Sight* (10), The Truth and Reconciliation (TRC) (39), and the National Inquiry into Missing and Murdered Indigenous women, girls, Two-Spirit and LGBTQQIA + reports (40). It is critical to draw on the guidence of these foundational reports to investigate access to routine healthcare and build upon these reports by looking at access among marginalized Indigenous cisgender and transgender women and Two-Spirit Peoples (8).

Indigenous women, Two-Spirit Peoples, sex workers, women living with HIV, and people with marginalized and minoritized gender identities live in the intersections of multiple forms of structural violence (5, 9, 41). As a result of the intersectional stigma (i.e., the convergence of multiple stigmatized identities among a group or person) and violence, health and health service inequities are produced and reproduced (5, 10, 31, 42). Previous research has emphasized the roles of social determinants of health such as violence and cultural safety and access to healthcare services and supports among Indigenous women, Two-Spirit Peoples, sex workers and women living with HIV (7, 31, 43–45). Marginalized Indigenous women and Two-Spirit Peoples are targets of colonial violence and acts of genocide that are supported by colonial structures (40). Despite Indigenous women accounting for approximately 4% of the population, Indigenous women are overrepresented among women experiencing gender-based violence, with Indigenous women accounting for 75% of the overall population of women experiencing violence (46–49). Indigenous women and Two-Spirit People who are pregnant face severe amounts of racism and violence in the medical system, including threats and actions of child apprehension (5, 50), birth alerts (51), racism (10), and reproductive violence through forced sterilization which impacts trust in the medical system for Indigenous women and Two-Spirit Peoples who are pregnant or caring for a child (51). Racism and violence are known to undermine the health and safety of Indigenous peoples and undermine their access to care (5). For example, while Indigenous Peoples make up 14% of the population in Winnipeg, Canada, one-fifth of homicides were Indigenous women in 2022 (52). Previous studies describe high prevalence of violence perpetrated across community, intimate partner and community contexts among sex workers (31, 53, 54), with a disproportionate burden among Indigenous sex workers and Two-Spirit Peoples in Canada (31, 53, 55). Women living with HIV and sex workers also face several barriers to accessing healthcare services including violence (42, 56). Sex workers face disproportionate health and social inequities, for example, high rates of HIV and STI’s, violence, and criminalization (56). Sex workers and women living with HIV face several sources of structural violence, including lifetime exposure to violence, intimate partner violence, gender-based violence, and police harassment that impacts their access to healthcare services (22, 31, 57). A growing body of evidence has highlighted how culturally safe and trauma-informed healthcare services may reduce barriers to healthcare services among racialized and minoritized populations (7, 58, 59). The *In Plain Sight and National Inquiry into Missing and Murdered Indigenous women, girls, Two-Spirit* and *LGBTQQIA +* reports call us to address barriers to access to health services and the urgent need for culturally safe care (10, 40).

There is a need to understand how the COVID-19 pandemic has impacted social and structural health inequities for marginalized Indigenous cisgender and transgender women and Two-Spirit Peoples. Historical and ongoing colonial violence has impacted Indigenous health and marginalized Indigenous women and Two-Spirit Peoples face multiple barriers to accessing reliable healthcare services (7). The impacts of the COVID-19 pandemic have left gaps in knowledge that are not only essential to address now but also in preparation for future pandemic planning in the health system. The aim of this study was to evaluate access to culturally safe health services, experiences of violence, changes in mental health and changes in routine healthcare access during the COVID-19 pandemic. We hypothesized that these factors would be associated with experiencing difficulty accessing routine healthcare in a cohort of marginalized Indigenous cisgender and transgender women and Two-Spirit Peoples in Metro Vancouver, Canada during the COVID-19 pandemic.

## Methods

### Study Design

This study is nested within a larger project called AMPLIFY (PI: Bingham). AMPLIFY is a community-based participatory action program of Indigenous research that aims to privilege Indigenous community-based voices to directly inform culturally safe and equitable health and justice for Indigenous women, gender diverse, and Two-Spirit Peoples. In partnership with the Vancouver Coastal Health Director of Indigenous Women and Family Health this work is guided by the Indigenous Matriarch Advisory Council (MAC) which consists of Indigenous Matriarchs, Elders, researchers, and community members.

Data for this study were drawn from two ongoing community-based prospective cohorts of marginalized women’s health and access to care on the ancestral, occupied territories of the Musqueam, Squamish, and Tsleil-Waututh Peoples in what is now referred to as Vancouver, BC: An Evaluation of Sex Workers’ Health Access (AESHA) and Sexual Health and HIV/AIDS: Women’s Longitudinal Needs Assessment (SHAWNA). AESHA was developed in 2010 based on extensive community collaborations with sex work agencies and is monitored by a Community Advisory Board. The SHAWNA cohort began in 2014 and operates as a partnership that includes women’s HIV and community services providers. SHAWNA is also guided by the Community Advisory Board and the Positive Women’s Advisory Board (5, 60). Eligibility for AESHA and SHAWNA includes identifying as a cisgender or transgender women[4] and being 14 years of age or older. Additional eligibility for AESHA includes exchanged sex for money within the last 30 days (i.e., active engagement in sex work), whereas SHAWNA eligibility includes being diagnosed with HIV and living in and/or accessing healthcare services in Metro Vancouver.

Participants completed baseline and semi-annual questionnaires that included detailed measures of socio-demographics, work and living environments, and healthcare access; additionally, both AESHA and SHAWNA asked detailed questions regarding impacts of the COVID-19, including self-reported impacts of how the pandemic impacted health access, work environment, housing and economic factors, violence, policing, and social outcomes. Informed consent was obtained for AESHA and SHAWN participants. Due to challenges connecting with marginalized participants amid COVID-19 pandemic response measures (i.e., lockdowns, closures), the sample of Indigenous sex workers and women living with HIV represents a sub-sample of Indigenous participants from the AESHA and SHAWNA cohorts. All participants received an honorarium of $65 CAD at each bi-annual visit and an additional $20 for completion of the COVID-19 supplementary questions. Questionnaire visits were administered by community interviewers with extensive lived and/or professional experience working with community members.

### Analytical Approach

This research is in line with the *Grandmothers Perspective* on disaggregated data, upholding disaggregated data as a tool of care to address inequities that are faced by Indigenous Peoples in Canada (61). The *Disaggregated demographic data collection in British Columbia: The grandmother perspective* report calls for data to be used to advance human rights and for data to be used to address systemic inequities (61). This research is centered around the calls to action and justice from foundational reports (10, 39, 40) that calls for research addressing health inequities such as access to care for Indigenous cisgender and transgender women and Two-Spirit Peoples. Decolonizing health research involves evaluating, reflecting, and working to dismantle structures that support colonization and racism against Indigenous Peoples. Variables were chosen based on Indigenous frameworks. Variable interpretation drew from Indigenous frameworks, intersectional stigma, and social determinants of health of sex workers recognizing factors that impact peoples lived experiences such as laws, policies, racialization, and health environments (62–65).

### Data Analysis

Analyses were restricted to participants who self-identified as Indigenous (e.g., First Nations, Métis, and Inuit) by answering “yes” to the question “Do you identify as an Aboriginal person, that is, First Nations, Métis, or Inuit?”.

#### Variables

Exposure and outcome variable are time-updated variables measured semi-annually, capturing current and past six months occurrences at each study visit, unless otherwise specified. Indigenous ancestry, gender identity, and sexual orientation are treated as time-fixed variables.

The primary outcome variable, “difficulty accessing a family doctor, nurse, or clinic for routine healthcare during the COVID-19 pandemic”, was a binary variable based on the question, “General changes related to your access to health and social supports” The following response options were coded as yes: Canceled or reduced services; Not able to or don’t know how to access telehealth or other virtual, phone, or online services; Family doctor, nurse, or clinic doesn’t have these virtual, phone, or online services; Less time to devote to people’s concerns; Afraid to use or avoid using due to fear of getting sick; And relevant other responses. Participants who did not report any of these options or who did not have a family doctor, nurse, or location for accessing routine healthcare were coded as no.

Based on existing evidence on access to routine healthcare services among Indigenous women, Two-Spirit[5] Peoples, sex workers, and women living with HIV, key individual (age, income) and structural (violence, criminalization, safety) explanatory variables were considered for inclusion in multivariable analysis. Individual and demographic variables included age (continuous, in years), Indigenous identity (First Nations, Metis, Inuit), accessed Canada Emergency Response Benefit (CERB) (governmental COVID-19 economic relief support(15)), gender identity (gender minority at any study visit, inclusive of trans [transgender, transsexual, other transfeminine identity], gender nonbinary [nonbinary, genderqueer], and/or Two-Spirit Indigenous women vs. cisgender at all study visits), sexual orientation (sexual minority identity at any study visit [inclusive of lesbian, gay, bisexual, asexual, queer, and Indigenous Two-Spirit women vs. heterosexual at all study visits]), Two-Spirit identity, self-rated health as good, history of pregnancy (in lifetime), living with HIV, exchanged sex for money, goods, and services (in last six months), and lifetime mental health diagnosis. Structural variables included any physical/sexual violence by any perpetrator (in lifetime and in the last six months), non-injection or injection drug use in last six months (excluding alcohol and cannabis), and history of incarceration in lifetime (defined as in jail overnight or longer). Self-reported impacts of the COVID-19 pandemic in the last six months included experiencing changes or concerns related to psychological/emotional well-being, community safety/violence, and lacked access to culturally safe health or social services.

### Statistical Analyses

Analyses were restricted to 143 participants who self-identified as Indigenous (First Nations, Métis, and Inuit) at baseline and who participated in AESHA and SHAWNA and answered the COVID-19 supplementary questionnaire from October 2020 to August 2021. Explanatory variables were stratified by the outcome and differences were compared using Pearson’s chi-squared test for categorical variables (or Fisher’s exact test where cell counts were small) and the Wilcoxon rank-sum test for continuous variables. Factors hypothesized to be associated with difficulty accessing routine healthcare during the COVID-19 pandemic were assessed using bivariate logistic regression using generalized estimating equations (GEE). Variable selection for the multivariable model was based on theoretical considerations as well as findings of bivariate analysis (p < 0.10). Statistical analyses were performed in SAS version 9.4 (SAS, Cary, NC). Odds ratios (OR) and adjusted odds ratios (AOR) are reported with 95% confidence intervals (CI), and all p-values are two-sided.

## RESULTS

Analyses included 142 participants who contributed a total of 199 observations between October 2020 to August 2021. During the study period, 27.5% reported experiencing difficulty accessing a family doctor/nurse/clinic for routine healthcare during the COVID-19 pandemic.

Baseline demographics (Table 1) indicate the median age of participants was 46 (IQR: 37–53). 73.3% (n = 104) identified as First Nations, 14.1% (n = 20) identified as Metis, 7.0% (n = 10) identified as having both First Nation and Metis ancestry, and 5.6% (n = 8) self-identified as Indigenous but did not specify their Indigenous ancestry. One-fifth (19.7%, n = 28) reported identifying as a gender minority (i.e., intersex, transexual, genderqueer, non-binary, other), over half (52.8%, n = 75) identified as a minority sexual orientation (i.e., gay, lesbian, bisexual, asexual, queer, Two-Spirit, other), and 16.2% (n = 23) identified as Two-Spirit. 78.2% (n = 111) had been pregnant at least once, 46.5% (n = 66) were living with HIV, 41.6% (n = 59) exchanged sex for money, goods and services, and 75.4% (n = 107) had a previous mental health diagnosis. With regards to criminalization and violence, 88.7% (n = 126) had been incarcerated in the past, 95.8% (n = 136) had experienced violence by any perpetrator (e.g., clients, intimate partners, others) in the past, and 20.4% (n = 29) had experienced violence by any perpetrator in the last 6 months. With regards to important self-reported experiences and impacts of the COVID-19 pandemic, 70.4% (n = 100) of participants reported negative changes to psychological and emotional well-being, 15.5% (n = 22) had concerns regarding safety or violence in their community during the COVID-19 pandemic, 4.9% (n = 7) lacked access to culturally safe health or social services and 51.4% (n = 73) had accessed emergency economic relief support (CERB).

In unadjusted bivariate GEE analysis (Table 2), factors associated with increased odds of difficulty accessing a family doctor, nurse, or clinic for routine healthcare over the study period included history of pregnancy (OR: 2.74; 95% CI: 0.93–8.10), accessed CERB (OR: 0.67; 95% CI: 0.33–1.36), and minority sexual orientation (OR: 1.57; 95% CI: 0.77–3.18) while injection drug use was associated with reduced odds of difficulty accessing routine healthcare (OR: 0.54; 95% CI: 0.23–1.25). COVID-19 factors associated with increased odd of difficulty accessing a family doctor, nurse, or clinic for routine healthcare included reporting having experienced negative impacts on psychological and emotional well-being (OR: 2.72; 95% CI: 1.11–6.69), lacked access to culturally safe health or social services (OR: 3.12; 95% CI: 0.99–9.79), and increased concerns regarding community safety/violence (OR: 2.57; 95% CI: 1.09–6.06).

In multivariable GEE analysis (Table 2), participants who had a history of pregnancy (AOR: 4.71, 95% CI: 1.33–16.66) had higher odds of facing difficulty accessing routine healthcare during COVID-19. COVID-19 factors associated with increased odds of difficulty accessing routine healthcare included having experienced negative changes in psychological and emotional well-being (AOR: 3.99, 95% CI: 1.33–11.98), lacked access to culturally safe health services (AOR: 4.67, 95% CI: 1.43–15.25), and concerns regarding community safety/violence (AOR: 2.72, 95% CI: 1.06–6.94).

## Discussion

This study aimed to evaluate factors associated with experiencing difficulty accessing routine healthcare among a cohort of marginalized Indigenous cisgender and transgender women and Two-Spirit Peoples in Metro Vancouver, Canada early in the COVID-19 pandemic (October 2020- August 2021). Among 142 participants, 27.5% reported difficulty accessing a family doctor, nurse, or clinic for routine healthcare. In this paper, we, as a team of Indigenous and allied scholars, demonstrate the need for accessible, culturally safe, gender-inclusive, and trauma-informed healthcare.

To evaluate potential inequities in access to routine healthcare among sexual minorities and gender-diverse Indigenous Peoples, we explored access to routine healthcare among participants with diverse gender identities, sexual orientation, and Two-Spirit identity. Of the thirty participants who identified that they had difficulty accessing routine healthcare during the COVID-19 pandemic, two-thirds identified as a minority gender identity, sexual orientation, and, or Two-Spirit. Previous research has highlighted increased difficulties in accessing primary healthcare services among Two-Spirit, non-binary, transgender, bisexual, gay, and queer people during the COVID-19 pandemic (8, 24, 28, 29).

This study also identified a high prevalence of physical and sexual violence and a strong association between difficulty accessing routine healthcare and concerns regarding community safety and violence during the COVID-19 pandemic. Almost our entire sample had experienced physical and or sexual violence by any perpetrator in the past, and 20.4% experienced violence by any perpetrator in the last six months. Previous research has highlighted the roles of structural factors such as violence that impact marginalized and criminalized women’s access to healthcare services (9, 31, 66). Our findings are consistent with a strong body of literature showing that Indigenous women and Two-Spirit Peoples face inequities that is rooted in structural racism and violence that is shaped by historical and ongoing colonization (5, 31, 67, 68). Urban poverty which stems from income inequalities, expansion of the criminal justice system in areas more populated with racialized and marginalized populations, and spatial segregation, disproportionately impacts people marginalized by social and structural inequities, including, Indigenous Peoples, sex workers, women living with HIV, people who use drugs, and houseless persons (69). In our study, marginalized Indigenous women and Two-Spirit Peoples who had concerns regarding safety or violence in community was associated with difficulty accessing routine healthcare. Characteristics of neighborhoods, including, community safety and violence, are known to influence a person’s access to health services (70–72). Prior spatial epidemiological research found that the spatial clustering of violence, community harassment, and policing, may displace marginalized sex workers to unsafe and unfamiliar areas where they may face increased barriers to healthcare (73). A study in the US, found that perceived community violence is associated with less routine healthcare utilization, highlighting the importance of community safety (74). Previous studies describe high prevalence of violence perpetrated across community, intimate partner and community contexts among sex workers (31, 53, 54), with a disproportionate burden among Indigenous sex workers, as well as among Two-Spirit and Indigenous Peoples with marginalized and minoritized sexual and or gender identities in Canada (31, 53, 55), who have also reported increased community and sexual violence during the COVID-19 pandemic (26, 75). Study findings demonstrate the need for community-based strategies to support safe and uninterrupted access to routine healthcare.

In our study, a high proportion (70.4%) of participants reported experiencing negative changes to psychological and emotional well-being during the COVID-19 pandemic, and this was associated with almost four-fold higher odds of difficulty accessing routine healthcare. Our findings are in line with previous research showing high levels of psychological and emotional stress during the COVID-19 pandemic (6, 18, 25–27), and in particular a high burden of stress, anxiety and depression among Indigenous Peoples in Canada during COVID-19 (18). A study in Canada among transgender and non-binary populations showed that poor mental health was associated with higher odds of avoiding primary care when compared to those who rated their mental health as good or excellent (29). Co-occurring social and public health crises such as COVID-19 and the ongoing overdose crisis sweeping the province of BC are likely to have long term impacts on marginalized Indigenous women and Two-Spirit Peoples. Women living with HIV have reported increased stress, anxiety and isolation as well as increased difficulty accessing HIV care during COVID-19 (27). Two-Spirit and Indigenous Peoples with marginalized and minoritized sexual and or gender identities in Canada have also reported concerns around deteriorating mental health during the COVID-19 pandemic (26). Marginalized Indigenous women and Two-Spirit Peoples face intersecting and compounding forms of oppression, and psychological and emotional stress further impacts their access to routine healthcare services.

In our study, marginalized Indigenous women and Two-Spirit Peoples who had a history of pregnancy faced increased difficulty in accessing routine healthcare. Our association between pregnancy and difficulty accessing routine healthcare may have occurred due the considerable disruptions to healthcare services, including routine, primary, and prenatal care access during the COVID-19 pandemic as well as the fear of being infected with the virus (76–78). When compared to non-Indigenous populations, Indigenous Peoples are less likely to access health care services due to persistent discrimination and medical racism (7, 37). Difficulty accessing routine healthcare that is trusted and safe may occur for Indigenous women and Two-Spirit People who are pregnant because of the severe amounts of racism in the medical system, including threats and actions of child apprehension (5, 50), racism, and reproductive violence through forced sterilization (51). Previous research has shown that unsafe and racist medical environments as well as social determinants of health impact access to pre-natal and post pregnancy care for Indigenous women, Two-Spirit Peoples, sex workers, and women living with HIV (21, 79–82). Most Indigenous Peoples in BC still cannot birth in their communities and there are striking inequities in access to birthing facilities for Indigenous Peoples (83, 84). There is limited research on factors that impact access to healthcare services for Indigenous parents and more research is needed to understand what access to healthcare looks like for Indigenous People who are pregnant and or parenting.

Our study showed that marginalized Indigenous women and Two-Spirit Peoples who lacked access to culturally safe health services was associated with greater odds of difficulty accessing routine healthcare. Canadian healthcare systems are not tailored to the needs of Indigenous communities and are often culturally, emotionally, and physically unsafe (26). Previous research has shown that healthcare services and systems that are not culturally safe can reduce access and engagement in health services (59). Access to culturally safe healthcare is particularly important for transgender populations, which have reported avoiding care due to discrimination faced when accessing routine healthcare (29). Cultural safety is the recognition of power imbalances and inequities within the healthcare system and focuses on creating safe environments free of racism and discrimination (85). Culturally safe environments create space and respect for a diversity of knowledge and understandings of health and well-being. The *In Plain Sight* report calls us to address individual and systematic racism in our health systems, issues that have arisen during COVID-19, access to health services, and the urgent need for culturally safe care (10). In line with the *In Plain Sight* and the TRC recommendations, Indigenous Peoples and researchers, have called for culturally safe services that protect the well-being of marginalized Indigenous women and Two-Spirit Peoples (8, 39, 86).

### Implications and Directions for Future Research and Programs

The current study further confirmed the need for culturally safe care for marginalized Indigenous women (cisgender and transgender) and Two-Spirit Peoples that is anti-racist, client-centered, trauma-informed, sex work informed, and gender inclusive. Access to healthcare spaces needs to extend beyond physical accessibility, considering social and structural factors that impact access to healthcare spaces and services, including concerns for violence in one’s neighbourhood (87). Indigenous healthcare workers significantly impact culturally safe spaces through addressing biases and providing cultural connection (88). Doctors, nurses, and healthcare providers in clinics need to be educated to provide culturally safe, anti-racist, trauma-informed, sex work informed, and gender inclusive healthcare services (89). There is an urgent need to address mental health service access to address the negative psychological and emotional impacts of the pandemic and to the improve the health of marginalized Indigenous women and Two-Spirit Peoples beyond COVID-19. Mental health services need to be enhanced to ensure these services are appropriate and culturally safe, for example, the inclusion of Elders and ceremony spaces in healthcare settings has been shown to create safer and more culturally appropriate environments for Indigenous Peoples (34, 45). Inequities in Indigenous reproductive rights have been historically colonial, and it is crucial to identify how Indigenous women and Two-Spirit Peoples can be supported during and beyond COVID-19 to access equitable and culturally safe sexual and reproductive health services. While this study highlighted structural and COVID-19 factors that impacted access to routine healthcare among Indigenous women and Two-Spirit Peoples, Indigenous perspectives and voices need to be prioritized and at the forefront in the creation of accessible and culturally safe health services. Addressing the anti-Indigenous racism that continues to ensure that Indigenous Peoples are intentionally excluded from accessing equitable healthcare means that Indigenous women, gender diverse, and Two-Spirit voices need to be prioritized in envisioning what cultural safety looks like in healthcare spaces. Indigenous Peoples are the best decision makers for their health and there needs to be more leadership positions in healthcare and health policy that allow Indigenous Peoples to speak about their own reality and experiences and to have the equitable and safe healthcare that they deserve. The inclusion of host Nations in creating accessible and culturally safe health care is vital in ensuring that we are respecting and honouring the land that the healthcare spaces are on.

## LIMITATIONS

The study has some notable limitations. First, the COVID-19 follow-up questionnaire was added shortly after the pandemic started in 2020. Due to the urgent need to address the public health crisis, only two cycles of data were collected and analyzed for this study. Second, because we restricted the analysis to only include people who self-identified as Indigenous and completed the COVID-19 questionnaire, the sample is small which limits statistical power and reduces the precision of estimates. This study took a unique approach to examine an only Indigenous cohort. Self-reported data are often subject to recall and social desirability bias, though our frontline staff includes experiential and community-based interviewers, which has been an effective strategy in mitigating this as much as possible. Due to the nature of quantitative data and study design, we can only analyze numerical relationships and associations between variables. This study highlights social and structural inequities that impact access to routine healthcare among marginalized Indigenous cisgender and transgender women and Two-Spirit Peoples. While taking a unique approach, this study only highlights difficulties in access to routine healthcare without discussing what access means and could look like among this group of people, however this study adds to the limited body of research on access to routine healthcare among marginalized Indigenous cisgender and transgender women and Two-Spirit Peoples. Future research is needed to address the lack of culturally safe care and to create community-led actionable changes within the Canadian healthcare system. While this study utilized community driven data and was guided by and lead by Indigenous Peoples future research needs to engage with marginalized Indigenous women and Two-Spirit Peoples to create actionable changes to address ongoing racism against Indigenous Peoples in the healthcare system and within Canada.

## CONCLUSION

This study aimed to evaluate factors associated with experiencing difficulty accessing routine healthcare among a cohort of marginalized Indigenous cisgender and transgender women and Two-Spirit Peoples in Metro Vancouver, Canada early in the COVID-19 pandemic. Approximately, one-quarter of our sample had difficulty accessing routine healthcare. Marginalized Indigenous women and Two-Spirit Peoples who had a history of pregnancy, experienced negative changes in psychological and emotional well-being, experienced community-based violence, and lacked access to culturally safe services, during the COVID-19 pandemic, had higher odds of difficulty accessing routine healthcare during the pandemic. Cultural approaches to health and wellness are diverse, and amplifying the voices of marginalized Indigenous women and Two-Spirit Peoples is vital to creating equitable health systems, services, and policies. Anti-racist and culturally safe health strategies and policies are needed at all levels of government to create safe, equitable, accessible health care for Indigenous Peoples (87). Findings are in line with the *In Plain Sight* recommendation to increase culturally safe health services and for the establishment of a system-wide measurement framework on Indigenous cultural safety, Indigenous rights to health, and Indigenous-specific racism (10). As we continue to unpack the impacts of historical and ongoing colonial violence on Indigenous Peoples, this research calls for the amplifying of Indigenous voices and informs actions to support culturally safe and antiracist health services, social well-being, and justice for marginalized Indigenous cisgender and transgender women and Two-Spirit Peoples.

## Figures and Tables

**Table 1:** Baseline demographic and structural characteristics amongst marginalized urban Indigenous women, gender diverse, and Two-Spirit Peoples, stratified by difficulty accessing family doctor/nurse/clinic for routine healthcare in Metro Vancouver, Canada, 2020–2021 (n = 142)

Characteristic	Total (%) (*n* = 142)	Difficulty accessing routine healthcare*		*p*-value
		Yes (%) (*n* = 30)	No (%) (*n* = 112)	
**Individual and Demographic Factors**				
Age (med, IQR)	45.5 (37–53)	42 (36–50)	47 (38–53)	0.269
Indigenous Identity/Ancestry				
First Nation	104 (73.3)	18 (60.0)	86 (76.8)	0.196
Metis	20 (14.1)	7 (23.3)	13 (11.6)	
First Nation & Metis	10 (7.0)	2 (6.7)	8 (7.1)	
Indigenous unspecified	8 (5.6)	3 (10.0)	5 (4.5)	
Women living with HIV	66 (46.5)	15 (50.0)	51 (45.5)	0.663
Exchanged sex for money, goods, and services*	59 (41.6)	10 (33.3)	49 (34.8)	0.367
Mental Health Diagnosis	107 (75.4)	24 (80.0)	83 (74.1)	0.506
Non-injection drug use*	73 (51.41)	15 (50.0)	58 (51.8)	0.901
Injection drug use*	41 (28.9)	6 (20.0)	35 (31.3)	0.295
Any drug use*	85 (59.9)	17 (56.7)	68 (60.7)	0.958
Minority gender identity	28 (19.7)	3 (10.0)	25 (22.3)	0.132
Minority sexual orientation	75 (52.8)	19 (63.3)	56 (50.0)	0.194
Two-Spirit identity	23 (16.2)	3 (10.0)	20 (17.9)	0.408
Minority gender identity and/or sexual orientation and/or Two-Spirit	80 (56.3)	19 (63.3)	61 (54.5)	0.384
Pregnant ever	111 (78.2)	27 (90.0)	84 (750)	0.136
**Structural factors**				
Incarceration ever	126 (88.7)	28 (93.3)	98 (87.5)	0.523
Physical/sexual violence by any perpetrator	136 (95.8)	30 (100.0)	106 (94.6)	1.00
**Individual and Demographic Factors**				
Physical/sexual violence by any perpetrator*	29 (20.4)	8 (26.7)	21 (18.8)	0.439
**COVID-19 Variables**				
Negative changes to psychological/emotional wellbeing*	100 (70.4)	28 (93.3)	72 (64.3)	0.002
Positive changes to psychological/emotional wellbeing*	20 (14.1)	2 (10.0)	17 (15.2)	0.567
Self-rated health was good*	82 (57.8)	15 (50.0)	67 (59.8)	0.282
Lacked access to culturally safe health or social services*	7 (4.9)	4 (13.3)	3 (2.7)	0.037
Increased police/security presence*	8 (5.6)	3 (10.0)	5 (4.5)	0.366
Concerns regarding safety or violence in community*	22 (15.5)	10 (33.3)	12 (10.7)	0.008
Accessed Canadian Emergency Response Benefit (CERB)	73 (51.4)	13 (43.3)	60 (53.6)	0.470

All data refer to *n* (%) of participants unless otherwise specified

* In the last 6 months

**Table 2:** Bivariate generalized estimating equations (GEE) analysis of correlates of difficulty accessing family doctor/nurse/clinic for routine healthcare among marginalized urban Indigenous women, gender diverse, and Two-Spirit Peoples in Metro Vancouver, Canada, 2020–2021 (n = 142)

	Unadjusted OR (95% CI)	Adjusted OR** (95% CI)
**Individual and Demographic Factors**		
Age (per year older)	1.01 (0.97–1.05)	
Women living with HIV	1.54 (0.76–3.11)	
Exchanged sex for money, goods, and services*	0.64 (0.31–1.33)	
Mental health diagnosis^‡^	0.91 (0.43–1.93)	
Non-injection Drug use*	1.27 (0.60–2.68)	
Injection Drug use*	**0.54 (0.23–1.25)**	
Any Drug Use*	1.16 (0.53–2.534)	
Minority gender identity	0.61 (0.25–1.50)	
Minority sexual orientation	1.57 (0.77–3.18)	
Two-Spirit identity	0.58 (0.22–1.55)	
Minority gender identity and/or sexual orientation and/or Two-Spirit	1.47 (0.72–3.01)	1.58 (0.74–3.40)
Pregnant ever	**2.74 (0.93–8.10)**	**4.71 (1.33–16.66)**
**Structural factors**		
Physical/sexual violence by any perpetrator*	0.99 (0.39–2.50)	
Incarceration ever	1.99 (0.42–9.37)	
**COVID-19 Variables**		
Negative changes to psychological/emotional well-being*	**2.72 (1.11–6.69)**	**3.99 (1.33–11.98)**
Positive changes to psychological/emotional well-being*	0.93 (0.32–2.67)	
Self-rated health was good	0.75 (0.40–1.41)	1.08 (0.51–2.26)
Lacked access to culturally* safe health or social services*	**3.12 (0.99–9.79)**	**4.67 (1.43–15.25)**
Concerns regarding safety or violence in community*	**2.57 (1.09–6.06)**	**2.72 (1.06–6.94)**
Increased police/security presence*	2.06 (0.58–7.35)	
Accessed Canadian Emergency Response Benefit (CERB)	0.67 (0.33–1.36)	
**Individual and Demographic Factors**		

* Time updated measure using the last six months as reference

## Data Availability

Due to our ethical and legal requirements related to protecting participant privacy and current ethical institutional approvals, de-identified data are available upon reasonable request and pending ethical approval. Please submit all request to initiate the data access process to the corresponding author.

## Footnotes

1. While the term Indigenous in used throughout this paper, we acknowledge the diversity and uniqueness among First Nations, Inuit, and Métis Peoples. Acknowledging and respecting the diversity in culture, traditions, and languages among and between First Nations, Inuit, and Métis groups, throughout this paper, the term Indigenous is used collectively to refer to the First Nations, Inuit, and Métis Peoples of Canada.

2. Two-Spirit (2S) identity is Nation specific and is an organizing strategy and or tool that is used to identify Indigenous Peoples from North America who embody diverse sexualities, genders, gender identities, and gender expressions.

3. The term ‘gender diverse’ is an umbrella term used to refer to people whose gender expression and or identity exists outside of a gender norm in a specific context. While gender diversity encompasses an ever-evolving array of labels, we honour the diversity within this general term.

4. Eligibility and recruitment criteria for AESHA and SHAWNA is inclusive of cis women, transgender women, transexual women and other self-reported transfeminine identities at enrolment. We acknowledge that gender identity is fluid and recognize that participants’ gender presentation and expression may differ throughout various times of their lives.

5. Two-Spirit identities and roles in community were drastically altered by historical and ongoing colonial violence. While Two-Spirit identity is only descriptively looked at in this study, we honour Two-Spirit roles within communities that were disrupted by colonialism and want to support the resurgence and well-being of Two-Spirit Peoples.
